# Cellular Plasticity in Breast Cancer Progression and Therapy

**DOI:** 10.3389/fmolb.2020.00072

**Published:** 2020-04-24

**Authors:** Deguang Kong, Connor J. Hughes, Heide L. Ford

**Affiliations:** ^1^Department of Pharmacology, University of Colorado Anschutz Medical Campus, Aurora, CO, United States; ^2^Department of General Surgery, Zhongnan Hospital of Wuhan University, Wuhan, China; ^3^Pharmacology Graduate Program, University of Colorado Anschutz Medical Campus, Aurora, CO, United States; ^4^Medical Scientist Training Program, University of Colorado Anschutz Medical Campus, Aurora, CO, United States

**Keywords:** breast cancer, plasticity, EMT, cancer stem cell, metastasis

## Abstract

With the exception of non-melanoma skin cancer, breast cancer is the most frequently diagnosed malignant disease among women, with the majority of mortality being attributable to metastatic disease. Thus, even with improved early screening and more targeted treatments which may enable better detection and control of early disease progression, metastatic disease remains a significant problem. While targeted therapies exist for breast cancer patients with particular subtypes of the disease (Her2+ and ER/PR+), even in these subtypes the therapies are often not efficacious once the patient's tumor metastasizes. Increases in stemness or epithelial-to-mesenchymal transition (EMT) in primary breast cancer cells lead to enhanced plasticity, enabling tumor progression, therapeutic resistance, and distant metastatic spread. Numerous signaling pathways, including MAPK, PI3K, STAT3, Wnt, Hedgehog, and Notch, amongst others, play a critical role in maintaining cell plasticity in breast cancer. Understanding the cellular and molecular mechanisms that regulate breast cancer cell plasticity is essential for understanding the biology of breast cancer progression and for developing novel and more effective therapeutic strategies for targeting metastatic disease. In this review we summarize relevant literature on mechanisms associated with breast cancer plasticity, tumor progression, and drug resistance.

## Introduction

With the exception of non-melanoma skin cancer, breast cancer is the most frequently diagnosed malignant disease among women (Bray et al., 2018). In 2018, there were about 2.1 million newly diagnosed cases worldwide (Bray et al., 2018). With the introduction of mammography coupled with improved treatment, breast cancer mortality rates have decreased 1.8 to 3.4% per year since 1990 (Hendrick et al., 2019). Nonetheless, breast cancer remains the leading cause of cancer death among females, claiming over 600,000 lives per year worldwide (Valastyan and Weinberg, 2011; Bray et al., 2018), with more than 90% of patients dying from metastatic disease (Valastyan and Weinberg, 2011). Currently, there are no effective treatment strategies for metastatic patients, regardless of breast cancer subtype, and the median overall survival remains at ~1–5 years (Waks and Winer, 2019). Therefore, understanding the cellular and molecular mechanisms that mediate cancer cell escape from the primary tumor, and most importantly, outgrowth and maintenance at secondary sites, is critical for developing novel therapies that specifically target metastatic disease.

**Figure 1:**
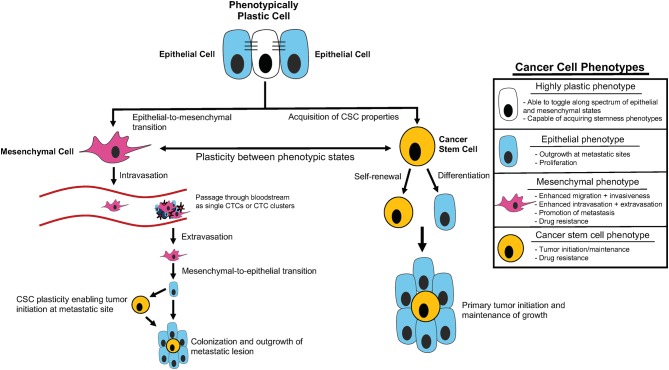
Cancer cell plasticity throughout tumor initiation and the metastatic cascade. Outline of contribution of epithelial-mesenchymal plasticity (EMP) and acquisition of cancer stem cell (CSC) properties to tumor initiation and components of the metastatic cascade, including intravasation, extravasation, and metastatic colonization/outgrowth. Summary of distinct cellular phenotypes and characteristics associated with each.

Metastasis is highly complex, requiring cells to adapt to numerous different microenvironments as they leave the primary site, invade into and disseminate through the vasculature, seed at a distant site, and finally colonize and expand to form macrometastases (Gupta and Massague, 2006; Micalizzi et al., 2017; Smigiel et al., 2019). To navigate all the steps of the metastatic cascade, tumor cells likely require significant plasticity (da Silva-Diz et al., 2018; Chu et al., 2019; Smigiel et al., 2019; Yuan et al., 2019) (Figure 1). Plasticity can be defined as the ability of cells to toggle between different phenotypes without altering genotype, and is widely observed in embryonic differentiation, wound repair, and cancer metastasis (Yuan et al., 2019). In part, plasticity may arise from a gain in progenitor or stem-like qualities and/or from induction of an epithelial-to-mesenchymal transition (EMT).

During development, stem cells with self-renewal capability generate progeny that differentiate into all the cells of the body (Thiery et al., 2009). Further, in the developing organism, a subset of epithelial cells undergo an EMT, and subsequently may undergo the reverse process (mesenchymal to epithelial transition [MET]), in order to enable epithelial sheets to fold and fuse to create the final shapes of the various tissues and organs (Thiery et al., 2009; Ray and Niswander, 2012). It has become increasingly appreciated that these developmental processes, which require cell plasticity, share commonalities with processes required for the progression of cancer (Ma et al., 2010; Manzo, 2019; Yuan et al., 2019). As the counterpart of normal stem cells, cancer stem cells (CSCs) are characterized by their ability to self-renew, in addition to their pluripotent and pro-tumorigenic properties (Mani et al., 2008; Morel et al., 2008). Similar to epithelial cells during development, carcinoma cells also perform EMT to become motile, enabling the spread of cells to distant organs (Thiery et al., 2009).

An association between EMT and cancer stem cells was first reported by Mani et al., where they demonstrated that transduction of human mammary epithelial cells with EMT transcription factors (Snail/Twist1) led not only to an increase in expression of mesenchymal markers and a phenotypic change toward mesenchymal morphology, but also led to an increase in the percentage of CD44^high^CD24^low^ cells with increased stemness properties (Mani et al., 2008). Other studies led to similar conclusions, where the induction of an EMT program in epithelial tumor cells increased the population of CSCs, enhancing their tumor initiation ability (Morel et al., 2008; Wellner et al., 2009). However, EMT cannot always be equated with cancer stemness. Nieto et al. demonstrated that the homeobox transcription factor Prrx1, which induced EMT and enabled invasiveness characteristics in a panel of human cancer cell lines, actually needed to be lost in order for breast cancer cells to metastasize *in vivo*. This loss of Prrx1 was associated with a reversion of EMT and induction of stem cell properties, suggesting that plasticity and EMT are not inextricably linked and the process of metastasis may require dynamic fluctuations between epithelial and mesenchymal states in cancer cells (Ocana et al., 2012).

In this review, we summarize current knowledge around cellular plasticity in breast cancer, specifically with regards to epithelial-mesenchymal plasticity (EMP) and plasticity of cancer stem cells, and the role of these processes in promoting tumor initiation, maintenance, and metastasis. We will also outline the impact of plasticity on drug resistance and explore recent findings in which targeting plasticity may be used to develop more effective therapeutic strategies.

## Cancer Stem Cell and EMT Plasticity in Breast Tumorigenesis

Breast cancer stem cells (BCSCs) are identified as a small population of cells that have specific molecular signatures such as CD44^+^/CD24^−^, Aldehyde dehydrogenase 1 high (ALDH1^high^), and CD133^+^ (Al-Hajj et al., 2003; Al-Hajj and Clarke, 2004; Butti et al., 2019). The origin of BCSCs is still controversial. Due to their ability to self-renew and to lead to differentiation when driving tumor growth, numerous researchers claim that BCSCs arise from mammary stem cells or progenitor cells (Liu et al., 2014; Bao et al., 2015; Sin and Lim, 2017). This claim is supported by the fact that BCSCs share specific cell markers and exhibit properties that are highly similar to normal mammary stem cells or partially differentiated mammary progenitor cells, such as self-renewal and long persistence in mammary tissue (Liu et al., 2014; Sin and Lim, 2017). In contrast to this hypothesis, other investigators argue that BCSCs can be derived from differentiated mammary cells. Indeed, several recent studies indicate that gene mutations, a damaging physical stimulus, or the tissue microenvironment can all transform differentiated cells into BCSCs (Lagadec et al., 2012; Chaffer et al., 2013; Koren et al., 2015). Thus, it is possible that BCSC may arise via several means, underscoring the plastic nature of cancer cells at various different stages of differentiation.

It is now well-understood that significant heterogeneity exists in tumors, and that only a subset of cells within primary breast tumors have tumor initiating potential. Because of the ability of BCSCs to self-renew and to also yield progeny that differentiate, a significant amount of research on these cells has revolved around their role in breast cancer initiation. The tumor initiating potential of BCSCs likely has important clinical relevance, as triple-negative breast cancer (TNBC), which has a higher population of BCSCs than other breast cancer subtypes, is more likely to relapse, providing an impetus for studies on this unique tumor cell population (Park et al., 2019).

In 2003, studies by Clarke and colleagues showed that breast tumor initiating stem cells (CD44^+^CD24^−/low^ lineage subpopulation) isolated from primary breast cancers could form tumors when transplanted into non-obese diabetic/severe combined immunodeficient (NOD/SCID) immunocompromised mice (Al-Hajj et al., 2003), however the remaining populations formed no detectable tumors even 29 weeks after injection into mice. Shortly after these studies, Dontu and colleagues discovered that ALDH1 could also mark BCSCs, and demonstrated that cells with high ALDH1 activity could generate mammospheres (a measure of anchorage-independent growth potential) *in vitro* and initiate tumors *in vivo* (Ginestier et al., 2007). Of note, different markers were used to define BCSC populations in these studies, and these markers do not identify the same populations. CD44^+^/CD24^−^ has been shown to mark mesenchymal-like CSCs, and ALDH1^high^ has been shown to mark epithelial-like CSCs (Liu et al., 2014). Importantly, BCSCs display plasticity between these epithelial and mesenchymal CSC states, with BCSCs expressing both markers simultaneously having the highest tumor initiating potential (Liu et al., 2014). These data suggest that stemness and EMP may coordinately regulate elements of tumor initiation and it is possible that these same characteristics are important not only for establishing primary tumors, but also for the initiation of metastatic lesions. Since those initial studies, additional studies have demonstrated even greater plasticity for BCSCs than originally anticipated. For example, BCSCs have been shown to be capable of differentiating into endothelial cells to support the formation of new blood vessels and further contribute to tumor growth (Delgado-Bellido et al., 2017). Therefore, tumor initiating potential is likely not the only way that highly plastic BCSCs can contribute to tumor progression.

A number of studies have suggested that cells that undergo an EMT (and thus are plastic in nature), are often more CSC-like, having gained self-renewal capabilities (May et al., 2011; Mallini et al., 2015; Yuan et al., 2019). In addition, conditions (such as hypoxia or addition of transforming growth factor beta) that induce EMT in human breast cancers also increase the proportion of CSCs, leading to increased resistance to chemotherapies and increased proliferation *in vitro*, as well as enhanced tumorigenicity *in vivo* (Mani et al., 2008; Shuang et al., 2014). As such, it has been proposed that some properties of tumor aggressiveness, including metastatic potential and therapeutic resistance, which have been attributed to CSCs, may also be due to activation of EMT programs in these cells (Gupta et al., 2019). Work by our group supports the connection between EMT and BCSCs by demonstrating that overexpression of the homeobox transcription factor, Six1, in a mammary gland-specific Six1-overexpressing transgenic mouse model increased the CSC pool while simultaneously producing tumors that exhibited a partial EMT phenotype (McCoy et al., 2009). Furthermore, several recent studies demonstrated that tumor-initiating ability of mesenchymal tumor-initiating cells was abolished when they were converted into epithelial counter parts (Avgustinova and Benitah, 2016; Chakraborty et al., 2016; Nilendu et al., 2018). These findings suggest contexts in which dynamic interplay between EMP and stemness can lead to distinct cancer cell populations with unique characteristics and activities.

However, while the tumor-initiating capacity of cancer cells may be dependent on the overall stemness of these cells, this stemness is not inextricably linked to an epithelial or mesenchymal state. A recent study by Weinberg et al. demonstrated that that hybrid epithelial/mesenchymal (E/M) breast cancer cells, which co-expressed both epithelial and mesenchymal markers, and were further defined by the antigen combination CD104^+^/CD44^hi^, were required for tumorigenicity. Mixing of cells expressing only epithelial or mesenchymal markers, respectively, did not recapitulate the tumorigenic potential of hybrid E/M cells which express both epithelial and mesenchymal markers simultaneously and likely represent an intermediate cell state with distinct phenotypic characteristics. Additionally, forcing hybrid E/M cells to a pure mesenchymal state through ectopic expression of Zeb1 abrogated the tumorigenic potential of these cells. This study suggests that the tumorigenic potential of CSCs may be more dependent on intrinsic cellular plasticity rather than EMT *per se* (Kroger et al., 2019).

With these studies in mind, it may be more appropriate to think of stemness and EMT as spectrums rather than distinct cell states, allowing for unique combinations of stem cell and E/M characteristics in a given subpopulation. Recent mathematical modeling approaches provide evidence for this line of thinking based on coupling of core decision-making modules of EMT (miR-200/ZEB) and stemness (LIN28/let-7) phenotypes. This modeling demonstrates that fine-tuning of the expression and interaction of these modules can alter the position of the “stemness window” on the “EMT axis” (Jolly et al., 2015). Additionally, these findings suggest that the position of the “stemness window” on the “EMT axis” is flexible and provides a unifying explanation for the seemingly contradictory connections between EMT, MET, and hybrid E/M states and stemness phenotypes (Jolly et al., 2015). Thus, it is possible that EMT and CSC phenotypes represent characteristics that define the overall EMP of a given cancer cell, and this plasticity may be the key driver of tumor progression related to EMT and cancer cell stemness (Ford and Thompson, 2010) (Figure 1).

## Stem Cell and EMT Plasticity in Progression and Metastasis

It is well-established that tumor-host cell interactions influence the growth and spread of breast cancer. Tumor metastasis and ultimate outgrowth are very complex processes which require tumor cells to navigate numerous different environments and undergo a multitude of obstacles to survival and growth. Thus, those cancer cells that are able to alter their characteristics in response to different environments are likely to best navigate the multiple steps of the metastatic cascade (Figure 1). Plasticity, as well as the ability to cooperate with neighboring tumor cells or cells in the microenvironment, contributes to successful metastatic dissemination, and is important to understand if we are ever to develop means to treat this disease or prevent deadly progression.

### Escape From the Primary Tumor Site and Intravasation Into the Vasculature

During breast cancer development (and the development of numerous carcinomas), a subset of tumor cells may undergo an EMT. This program can be initiated by EMT-associated transcription factors (often induced in response to microenvironmental signals) and results in decreased expression of E-cadherin, claudins, occludins, and other proteins that are key components of adherence junctions and desmosomes (Peinado et al., 2004; Thiery and Sleeman, 2006). Concomitant with the loss of epithelial proteins, mesenchymal-associated proteins such as N-cadherin, Vimentin, fibronectin, and α-smooth muscle actin, can become up-regulated. As a result, intercellular contacts and apical-basal polarity are lost, and tumor cell motility is enhanced via reorganization of the actin cytoskeleton and the intermediate filament network (Thiery et al., 2009; May et al., 2011). EMT-associated transcription factors can also stimulate secretion of gelatinases and matrix metalloproteinases (MMPs), leading to remodeling of the extracellular matrix (ECM) (Galindo-Hernandez et al., 2015; Shen et al., 2017). The induction of EMT in carcinomas can further increase tumor angiogenesis via enriching CSCs which possess the capacity to differentiate into endothelial cells and also upregulate the expression of the pro-angiogenic transcription factor VEGF-A (Fantozzi et al., 2014; Delgado-Bellido et al., 2017). Collectively, these changes disrupt the contiguity of the tissue epithelium and basement membrane and enable enhanced cancer cell motility, rendering the cells able to invade into bloodstream.

The existence of EMP in breast cancer has become evident both from animal model and human studies. Studies from our group demonstrated that overexpression of SIX1 in the breast cancer cell line MCF-7 converted transforming growth factor-ß (TGF-ß) signaling from tumor suppressive to tumor-promotional (Micalizzi et al., 2010), and this modification of TGF-ß signaling additionally promoted EMT and enhanced metastasis in both experimental and spontaneous mouse models (Micalizzi et al., 2009). In line with this data, and importantly in the setting of the human disease, Maheswaran and colleagues found that mesenchymal cells expressing known EMT regulators, including TGF-β pathway components and the FOXC1 transcription factor, were highly enriched in circulating tumor cells (CTCs) and these mesenchymal CTCs were associated with disease progression (Yu et al., 2013). Similarly, Agelaki and colleagues found that EMT markers (Twist and Vimentin) are expressed in CTCs of patients with metastatic disease and in early breast cancer patients (Kallergi et al., 2011). Additionally, Maheswaran and colleagues also noticed small populations of CTCs that were positive for both epithelial and mesenchymal markers by RNA*-in situ* hybridization, and these hybrid E/M CTCs were often enriched in patients with progressive disease after chemotherapy (Yu et al., 2013). In this same study, an index patient demonstrated dynamic switching between mesenchymal and epithelial CTCs upon each cycle of therapy, suggesting that CTCs may maintain dynamic E/M plasticity (Yu et al., 2013; Hinohara and Polyak, 2019). This data aligns well with a recent study from Gupta and colleagues which utilized a DNA barcoding approach in the human breast cancer cell line MDA-MB-157 in order to demonstrate that distinct clonal populations of tumor cells can fluctuate between epithelial and mesenchymal states, demonstrating intrinsic E/M plasticity (Mathis et al., 2017). Additionally, they further demonstrated that progeny from a single clonal population maintain stable epithelial-to-mesenchymal ratios, suggesting that there may be an intrinsic component of distinct tumor clones which define their overall tropism for epithelial or mesenchymal states (Mathis et al., 2017). In fact, it is possible that cells maintaining both epithelial and mesenchymal characteristics may be the most metastatic, as a recent study demonstrated that intravenous injection of mammary tumor subpopulations from different stages of EMT saw the strongest increase in metastatic potential of early hybrid E/M states (Gupta et al., 2019; Pastushenko and Blanpain, 2019). After sorting, the majority of CTCs observed after IV injection exhibited an EpCAM-CD106-CD51-CD61 phenotype which is associated with co-expression of epithelial and mesenchymal markers (Gupta et al., 2019; Pastushenko and Blanpain, 2019). This study provides evidence that metastasis may be more dependent on maintaining EMP and hybrid E/M characteristics than it is on cells undergoing a complete EMT. Additionally, this plasticity may extend to stemness as well, as CTCs isolated from patients with breast cancer or from xenografts derived from patients with breast cancer overexpress both EMT markers and stem cell markers (Aktas et al., 2009; Baccelli et al., 2013). Together, these data suggest that highly plastic cells are more likely to make it to or survive in the bloodstream and represent the primary pool of cells from which metastatic lesions arise.

It is hypothesized that these highly metastatic hybrid E/M cells and cancer stem cells may be generated in or maintain local signaling in and around the primary tumor, which spatially primes tumors to seed cells into the bloodstream. Mathematical modeling experiments demonstrate that concentration gradients of EMT inducing signals (such as TGF-β) from the tumor-stroma boundary can generate distinct spatial patterning within tumors where complete EMT cells cluster toward the invasive edge and hybrid E/M cells are generated closer to the interior of the tumor where the concentrations of these signaling molecules are lower (Bocci et al., 2019). This may, in part, explain why mesenchymal cells are highly enriched in CTCs (Yu et al., 2013). In this model, CSC properties are generated in both the pure EMT and hybrid E/M populations, suggesting that both of these cell populations are intrinsically plastic. Subsequent addition of inflammatory cytokine signaling to this model enhances Notch signaling and stabilizes cells within the hybrid E/M state, offering an explanation for how stable hybrid E/M cells may reach the periphery of the tumor, form clusters, and dislodge from the primary tumor and enter the circulation without undergoing a complete EMT (Bocci et al., 2019). These mathematical findings suggest that cancer cell plasticity likely enables or facilitates movement of cancer cells from the primary tumor into the vasculature and may enhance survival of these cells in the circulation during therapy.

### Plasticity as a Means to Survive in the Circulation

CTCs confront a harsh environment (shear stress, anoikis, and cytotoxic immune attack) making it difficult to survive as they move through the bloodstream, resulting in a large number of CTCs that are apoptotic in cancer patients (Francart et al., 2018). In breast cancer, Agelaki and colleagues found that a low percentage of apoptotic CTCs was associated with advanced clinical parameters, suggesting that having CTCs that are able to resist apoptosis may predict worse clinical disease (Kallergi et al., 2013). In this section, we will discuss how cellular plasticity contributes to survival of CTCs in the bloodstream.

Recent studies have demonstrated that undergoing an EMT in the initial invasion steps of metastasis may protect tumor cells from anoikis once in the bloodstream (Charpentier and Martin, 2013). For example, Weinberg and colleagues showed that loss of E-cadherin, one of the hallmarks of EMT, can enhance anoikis resistance of immortalized human mammary epithelial cells via inhibition of phosphorylation of β-catenin, thus stabilizing the protein by inhibiting its recognition by the proteasome (Onder et al., 2008). In an epithelium-specific p53 knock out mouse tumor model, Jonkers and colleagues similarly found that loss of E-cadherin could promote tumor metastasis by inducing increased anoikis resistance (Derksen et al., 2006). Furthermore, in breast cancer cell lines, loss of E-cadherin suppresses the activity of Ankyrin–NRAGE–p14ARF signaling to confer anoikis resistance (Kumar et al., 2011; Frisch et al., 2013). EMP and cancer stemness may act cooperatively to enhance anoikis resistance, as Frisch and colleagues showed that in breast cancer, the cancer stem cell marker CD44S, which is up-regulated in response to an EMT, can enhance cell survival under detached conditions (Cieply et al., 2015).

EMP affects numerous characteristics of tumor cells beyond the expression of epithelial and mesenchymal markers. For example, microtentacles, produced via dynamic microtubule-based extensions of the plasma membrane, have been shown to be critical for CTCs to resist shear stress and anoikis in circulation (Yamauchi et al., 2005; Charpentier and Martin, 2013). In human mammary epithelial cells or breast cancer cells, EMT-associated transcription factors, including Snail1 and Twist1, could up-regulate this cytoskeletal structure, and vimentin filaments, a known marker for EMT, supported extension of these microtentacles (Whipple et al., 2008, 2010).

As more studies on CTCs have been performed, it has become clear that CTCs can exist as single cells or in clusters containing mixes of tumor cells, immune cells, stromal cells, pericytes, platelets, and cancer-associated fibroblasts (Hong et al., 2016) (Figure 1). In breast cancer, CTC clusters have been shown to be more metastatic than single CTCs, and their presence is associated with a poor prognosis (Aceto et al., 2014). Blackhall and colleagues have demonstrated that when compared to single CTCs, tumor cells within CTC clusters exhibit enhanced survival and decreased anoikis (Hou et al., 2011). Emerging evidence suggests that EMT, and its reverse program MET, play important roles in the formation of CTC clusters. Maheswaran and colleagues showed that the formation of CTC clusters relies on the expression of the cell–cell adhesion molecule plakoglobin in a mouse mammary carcinoma model (Aceto et al., 2014). However, in that study, they did not explore the EMT status in these CTCs cluster cells. Cheung and colleagues found that Keratin 14, an epithelial cytoskeletal protein, is highly expressed in murine breast cancer CTC clusters and that these Keratin 14 positive cells exhibit a hybrid E/M phenotype expressing both epithelial and EMT/stemness mesenchymal markers (Cheung et al., 2016), again indicating that plasticity may be a key feature for survival of tumor cells that leave the primary tumor. In contrast, Blackhall and colleagues found that lung carcinoma cells within CTC clusters primarily remain very mesenchymal, expressing Vimentin but not expressing E-cadherin. In addition, they also found that the EMT status of CTC cluster cells was more pronounced than that of single CTCs (Hou et al., 2011). There are many considerations for why these studies yielded divergent findings. First, the Blackhall study utilized human lung cancer patient blood samples, whereas the Cheung study utilized a murine breast cancer model, and thus it's possible the murine model doesn't accurately portray human cancer biology or that lung and breast cancer may utilize different strategies for CTC dispersal and survival. It is important to note that the Blackhall study uncovered a high degree of intra- and inter-patient heterogeneity with regards to staining for epithelial markers in CTC clusters. Thus, loss of membranous E-cadherin may not represent a complete loss of the epithelial phenotype of these cells as other epithelial markers can be expressed in this context to promote aggregation of these clusters. While there remains much to be understood about CTC cell plasticity, these studies suggest that carcinoma CTCs can exist in various states on the spectrum from epithelial to mesenchymal, underscoring the benefits of plasticity in the process of metastasis.

Emerging evidence suggests that coagulation activated by CTCs also plays an important role in enhancing the ability of CTCs to survive in the bloodstream. Many kinds of tumor cells, including breast cancer cells, express tissue factor, which is an important cell-associated activator of the coagulation cascade (Palumbo, 2008; Cole and Bromberg, 2013; Lambert et al., 2017). Once the tumor cells invade into the circulation, CTCs rapidly associate with platelets to activate the coagulation cascade and form platelet-rich thrombi around tumor cells in the vasculature. These thrombi are thought to physically protect CTCs from the stress of blood flow and from elimination by the immune system (Labelle and Hynes, 2012). This idea has also been supported by clinical studies. In a variety of malignant diseases, coagulation has been associated with a poor clinical prognosis and anticoagulants can reduce metastasis (Lee, 2010; Degen and Palumbo, 2012). Further, Cristofanilli and colleagues found that CTCs in metastatic breast cancer patients are associated with increased risk of thromboembolism (Mego et al., 2009). Strikingly, a relationship between EMT and tissue factor has been observed in several cancers (including breast cancer), again suggesting that plasticity is a key factor in mediating this phenotype. For example, in breast cancer cell lines, cells induced to undergo an EMT via introduction of Zeb1 increased the expression of tissue factor, which led to increased coagulant properties. Silencing Zeb1 inhibited both EMT-associated TF expression and coagulant activity (Bourcy et al., 2016). Taken together, these studies, as well as many others, suggest that EMP may be a key means by which cells survive the early steps of metastasis.

### Tumor Cell Extravasation

After surviving in the circulation, CTCs must first attach to the capillary endothelium and then penetrate a physical barrier composed of an endothelial and pericyte cell layer to effectively develop into a metastatic lesion. Most circulating cancer cells become trapped in capillaries due to size restriction. Compared to single cells, cancer cell clusters are larger and travel more slowly, and can thus easily be trapped in small blood vessels in various organs (Yu, 2019). This entrapment and arrest may be one mechanism by which CTC clusters and CTC-containing-thrombi (regulated by EMT) promote metastases as this increased residence time may facilitate increased interaction with the endothelial wall and subsequent extravasation. In addition to a passive entrapment in the vasculature, under certain conditions cancer cells can undergo adhesive arrest in the capillary vessels that are larger than the cell diameter in an active manner (Yamauchi et al., 2005). Similar to leukocytes, CTCs can roll and adhere to endothelial cells. CTC clusters and CTC-containing- thrombi have a much lower rolling velocity, and are thus susceptible to increased interaction with the vascular wall (Francart et al., 2018). In addition, tumor cells express specific proteins such as selectins, integrins, and metadherin, which enable active adhesion to the vasculature (Orr and Wang, 2001; Brown and Ruoslahti, 2004; Wang et al., 2004; Labelle and Hynes, 2012). Weinberg and colleagues found that the EMT-associated transcription factors (Snail1 and Twist1), when expressed in murine mammary carcinoma lines, promote the formation of filopodia-like protrusions (FLPs) which contain integrin β1, enabling interaction with the ECM (Shibue et al., 2012). In contrast, Klemke and colleagues found that Twist1 expression in human breast tumor cells promoted tumor cell adherence to the vascular wall through a β1 integrin-independent mechanism (Stoletov et al., 2010). In addition, Twist1 positive cells formed large dynamic rounded membrane protrusions, promoting the ability for tumor cells to traverse capillary vessels (Stoletov et al., 2010). It is possible that the dependence or lack of dependence of this process on β1 integrin may depend on the species of origin or specific cell line, or that abundant expression of β1 integrin in murine FLPs doesn't necessarily imply that this expression is explicitly required for the adhesion function of these protrusions. In any case, it remains that EMT and cancer cell plasticity can facilitate extravasation through multiple, possibly synergistic, mechanisms.

### Colonization of Distant Organ Sites

The microenvironment of the secondary site is often very different from the primary site, creating a significant challenge for disseminated tumor cell (DTC) survival. In 1889, Steven Paget first proposed the ‘seed and soil’ hypothesis, which proposed the need for a receptive microenvironment for the growth of metastases (Paget, 1989). In recent years, this hypothesis has been supported by experimental studies, leading to the more recently described concept of a pre-metastatic niche. The pre-metastatic niche has been shown to be educated by tumor-derived secreted factors, extracellular vesicles, bone marrow-derived cells, suppressive immune cells and host stromal cells, in order to become a receptive microenvironment for DTC colonization (Liu and Cao, 2016). Strikingly, Cano and colleagues uncovered a relationship between EMT and the formation of a premetastatic niche in breast cancer (Canesin et al., 2015). Tumor cell expression of lysyl oxidase-like 2 (LOXL2) has been shown to regulate the EMT transcription factor Snail1 and can additionally interact with the bHLH transcription factor E47 to downregulate E-cadherin and induce EMT (Canesin et al., 2015; Salvador et al., 2017). In addition to regulating EMT-associated transcription factors, LOXL2 additionally regulates the recruitment of bone marrow progenitor cells (c-kit^+^/Sca-1^+^) to the lungs and enhances premetastatic niche formation, demonstrating multiple, simultaneous means by which tumor cells may enhance their metastatic potential (Canesin et al., 2015).

While EMP appears to be critical in the earlier stages of metastasis, cancer stemness, which is associated with self-renewal and tumor initiation, is another form of plasticity that is likely most important in metastatic colonization. For example, our laboratory demonstrated that SIX2 overexpression in breast cancer cells leads to efficient metastatic colonization in the lung via its ability to induce a CSC phenotype through upregulation of SOX2 (Oliphant et al., 2019). Wong and colleagues also demonstrated a critical role for CSCs in metastatic colonization (Ren et al., 2018). The authors obtained triple-negative breast cancer patient-derived and cell line–derived CSC- enriched populations via growth as tumorspheres. CSCs obtained from tumorspheres formed brain metastases more rapidly after intracardiac injection into mice than their origin cell lines. It was observed that maintenance of stemness in these CSCs in this model occurred through activation of a tumor cell PCDH7-PLCβ-Ca^2+^-CaMKII/S100A4 signaling axis. When stemness was inhibited through administration of a specific PLC inhibitor edelfosine which disrupted this axis, brain metastatic colonization was significantly decreased (Ren et al., 2018).

Recent studies suggest that EMT may only be critical for the initial steps of the metastatic cascade up to organ extravasation, while its reverse process, MET, is associated with the tumor-initiating ability required for metastatic colonization (Acloque et al., 2009; Thiery et al., 2009). This hypothesis is reinforced by histological examination in clinical specimens, as metastatic tumors exhibit epithelial characteristics that are similar to those seen in the primary tumors (Chui, 2013). Wells and colleagues showed mesenchymal-like MDA-MB-231 breast cancer cells re-express E-cadherin through loss of methylation in the E-cadherin promoter when the cells reach the secondary organ environment via tail vein injection (Chao et al., 2010). In a dynamic *in vivo* model of metastatic breast cancer, Gilles and colleagues found that tumor cells in vascular tumoral emboli all express Vimentin (a marker of mesenchymal cells), but macrometastases in the lung display heterogenous Vimentin expression, and thus resemble the primary tumor (Bonnomet et al., 2012). In addition, Lieberman and colleagues found that miR-200, which promotes an MET, enhances macroscopic metastases in mouse breast cancer cell lines (Dykxhoorn et al., 2009). But how does MET influence metastatic colonization, particularly when EMT has been associated with stemness? Somewhat counterintuitively, stemness caused by MET may be one of the reasons. Indeed, growing evidence supports that MET is also linked to stemness. For example, MET is required to reprogram fibroblasts to iPSCs (Li et al., 2010; Samavarchi-Tehrani et al., 2010). During the reprogramming process, Snail1, TGF-β1 and TGF-β receptor II are repressed, and E-cadherin is up-regulated (Li et al., 2010). In breast cancer, recent studies also indicate a relationship between MET and stemness. Benezra and colleagues showed that during metastatic colonization, inhibitor of differentiation 1 (Id1), enhances breast cancer cells' stem-like phenotype by suppressing Twist1 and inducing an MET (Stankic et al., 2013). While these findings seemingly contradict the relationship of EMT in promoting stemness (May et al., 2011; Delgado-Bellido et al., 2017; Yuan et al., 2019), the two ideas may be unified through the concept of the hybrid E/M state, which may be indicative of plasticity. As discussed previously, this concept is supported by mathematical modeling of EMT and stemness due to intrinsic signaling modules (Jolly et al., 2015) or concentration gradients of secreted signaling molecules in the tumor microenvironment (Bocci et al., 2019). Possibly as a consequence of MET, a subset of cells can be found in a hybrid E/M state (Grosse-Wilde et al., 2015). Therefore, it may be the case that a cell that has undergone an EMT and is more stem-like may also be more plastic and thus better able to undergo a MET or fluctuate along a spectrum of epithelial and mesenchymal states. Grosse-Wilde and colleagues demonstrated that in breast cancer, the hybrid E/M state reflects stemness and increased plasticity, as these cells demonstrate increased self-renewal, mammosphere formation, and can produce ALDH1^+^ progeny. Further, such hybrid cells are associated with poor prognosis (Grosse-Wilde et al., 2015). Weinberg and colleagues also demonstrate that the hybrid E/M state is essential for tumorigenicity of breast cancer (Kroger et al., 2019) further entwining the relationship between EMP and stemness. This theory was verified in ovarian and prostate cancer cells as well (Strauss et al., 2011; Ruscetti et al., 2015). Another possible means by which MET may induce colonization is through its ability to relieve the repression of proliferation caused by EMT. Nieto and colleagues found that during EMT, Snail impaired cell proliferation via repressing Cyclin D2 transcription (Vega et al., 2004). Tulchinsky and colleagues demonstrated that induction of EMT by Zeb1 directly repressed cell division by inhibiting Cyclin D1 activity (Mejlvang et al., 2007). Thus, there are a host of distinct mechanisms through which complete EMT may suppress and MET/EMP may facilitate outgrowth and establishment of metastatic lesions.

### Escape From Immune System

Evasion of the immune system is required if tumors are to recur or progress (Hanahan and Weinberg, 2011). Not surprisingly, cancer cell plasticity may be a key means through which tumor cells avoid detection by the immune system. Zhou and colleagues found that in cells enriched for BCSCs (ALDH^+^ or CD44+CD24^−^ cell populations), extracellular−5'- nucleotidase (CD73) was increased, which enzymatically produces extracellular adenosine and thus can activate adenosine signaling in immune cells. Adenosine signaling has been shown to suppress a variety of immune responses through a variety of distinct mechanisms including upregulation of the negative co-stimulatory molecules CTLA-4 and PD-1 in lymphocytes (Hasko et al., 2008; Zhang, 2010; Allard et al., 2013; Gajewski et al., 2013). These data suggest that BCSCs might promote breast cancer development and progression through immune evasion (Yu et al., 2017). In addition, Marcato and colleagues found that ALDH^+^ BCSCs had decreased expression of antigen processing and co-stimulatory molecules when compared to non-CSCs (Sultan et al., 2018). As a result, BCSCs could be less susceptible to T cell-mediated cytotoxicity.

Immunosuppressive effects of BCSCs extend to the innate immune system as well. Semenza and colleagues found CD47 expressed on BCSCs could enable cancer cells to evade phagocytosis by tumor-associated macrophages (TAMs) (Zhang et al., 2015). Additionally, Bian and colleagues demonstrated that BCSCs were resistant to the attack mediated by autologous/allogeneic NK cells due to reduced expression of MICA and MICB which were the ligands for the stimulatory NK cell receptor NKG2D (Wang et al., 2014). In addition to direct suppression of immune cells, BCSCs may also be capable of immune suppression through modulation of cytokine signaling in the tumor microenvironment. Farrar and colleagues found that BCSCs (CD44^+^/CD24^−^) express increased levels of CD200 on their cell surface (Kawasaki et al., 2007). CD200 is a member of the immunoglobulin superfamily involved in immunoregulation and tolerance. Its expression on ovarian and melanoma cancer cells was shown to suppress the anti-tumor immune response through downregulation of Th1 cytokines IL-2 and IFN-γ (Kawasaki and Farrar, 2008).

In tumor immune escape, there is a close relationship between EMT and cancer stemness. EMT can reduce immune detection as well as increase the percentage of cells with CSC characteristics. Chouaib and colleagues found that acquisition of the EMT phenotype in MCF-7 cells is associated with increased CD24^−^/CD44^+^/ALDH^+^ stem cell populations and is also associated with an inhibition of cytotoxic T lymphocytes (Akalay et al., 2013). This finding was strengthened by another study which found that immunoediting of breast tumor cells may be accompanied by both an EMT and the acquisition of a stem-like state in a neu-transgenic mouse model of breast cancer (Knutson et al., 2006). Further studies demonstrate that compared to non-CSCs, PD-L1 total protein and surface expression was enriched in BCSCs (CD44^+^/CD24^−/low^ population in human breast cancer and CD44^+^/CD24^+^/ALDH1^+^ population in mouse breast cancer) and this enrichment was regulated in response to EMT through an EMT/ß-catenin/STT3/PD-L1 signaling axis. EMT-induced ß-catenin transcriptionally upregulates the N-glycosyltransferase STT3, which N-glycosylates and subsequently stabilizes PD-L1 from degradation (Hsu et al., 2018). Intriguingly, although the induction of an EMT could upregulate PD-L1 on the surface Of non-CSC breast cancer cells, EMT led to a more robust PD-L1 induction in the BCSC populations (Hsu et al., 2018). In addition to up-regulation of PD-L1, down-regulation of MHC-I on the surface of breast cancer cells has been observed in response to EMT, protecting these cells and their more epithelial counterparts from immune attack (Dongre et al., 2017). These data indicate that there are multiple means through which EMT and cancer stemness can protect tumor cells from immune attack, and it is likely that tumor cells that have undergone an EMT and possess CSC properties confer even greater protection from immune clearance by simultaneously engaging multiple of the aforementioned immunosuppressive mechanisms.

## Influence of the Microenvironment and Tumor Cell Crosstalk on Breast Cancer Cell Plasticity

As described above, cancer cell plasticity is frequently regulated by dynamic cell-intrinsic EMT and stemness gene expression patterns. However, regulation of plasticity is also highly dependent on tumor cell-extrinsic microenvironmental influences. For example, an elegant *in vitro* study by Gupta and colleagues showed that in short-term 2D cultures, mammary epithelial cells spontaneously acquire stem-like traits. However, culturing of these same cells in 3D matrices more representative of *in vivo* tissue architecture preserves lineage identity (Sokol et al., 2016; Gupta et al., 2019). This finding suggests that tissue architecture may be capable of regulating cellular stemness, and this regulatory mechanism may also act to regulate generation or maintenance of BCSCs.

In addition to structural elements of the tumor microenvironment, local signaling between tumor cell subpopulations and between tumor cells and non-tumor cells can also influence cancer cell stemness, EMP, and tumor aggressiveness. Luo and colleagues demonstrated that tumor-associated macrophages (TAMs) were capable of paracrine activation of an EGFR-STAT3-SOX2 signaling axis in the 4T07 and 4T1 murine breast cancer cell lines leading to enhanced BCSC properties and tumor-initiating potential (Yang et al., 2013). Similarly, it was shown that estrogen could expand the BCSC pool in multiple human ER+ breast cancer cell lines through activation of a paracrine FGF/FGFR/Tbx3 axis in the cancer cell, greatly increasing tumorsphere formation potential of these cancer cells (Fillmore et al., 2010).

Recent work by our laboratory demonstrated that breast cancer cells that have undergone an EMT are capable of inducing EMT-like phenotypes and enhancing metastatic potential of non-EMT cells by activating GLI signaling in neighboring non-EMT cells (Neelakantan et al., 2017). In this way, signals from subsets of cells within a heterogeneous tumors could promote EMP and enhanced tumor metastasis in cells not intrinsically expressing EMT-associated transcription factors (Neelakantan et al., 2017).

Similar to stemness, EMP can also be induced through microenvironmental signaling driven by non-tumor cells. Feng and colleagues found that cancer-associated fibroblasts isolated from breast cancer tissues secreted TGF-β1, which was capable of activating TGF-β/Smad signaling in multiple breast cancer cell lines, leading to upregulation of EMT-associated transcription factors, and promoting an EMT phenotype (Yu et al., 2014). Similarly, Aboussekhra and colleagues demonstrated that SDF-1/MMP-2 secreted by cancer-associated fibroblasts deficient in p16 could induce an EMT in MDA-MB-231 breast cancer cells, again suggesting mechanisms of paracrine regulation of EMT in breast cancer (Al-Ansari et al., 2013).

Treatment with exogenous therapies also represents an extrinsic factor which can modulate cancer cell plasticity. An example of this was demonstrated by Gupta and colleagues, who used mathematical modeling approaches to simulate treatment of breast cancer cells with epithelial or mesenchymal-specific targeting drugs. This simulation suggested that sequential treatment of E or M specific therapies would lead to selection of plastic E/M clones with enhanced therapeutic resistance (Mathis et al., 2017). Similarly, it was demonstrated *in vitro* that radiation therapy led to a dose-dependent increase in BCSCs in single cell suspensions of human breast cancer specimens, as quantified by ALDH1 positivity (Lagadec et al., 2012). These examples demonstrate that while cancer cell plasticity often results from changes in cell-intrinsic gene expression and signaling, extrinsic effects of the microenvironment and tumor cell crosstalk also play a crucial role in regulating cancer cell stemness and EMP.

## Clinical Challenges Caused by Breast Cancer Cell Plasticity

The presence of intratumor heterogeneity, which describes the coexistence of cells that are genetically, epigenetically, or phenotypically different within the primary tumor or the metastatic site, creates a significant challenge for clinical diagnosis and therapy (Hong et al., 2018). Such heterogeneity can cause incorrect diagnoses or treatment when a small biopsy is used for pathological examination. Heterogeneity can also lead to resistance to chemotherapy and radiotherapy, as minor clones can be selected for during the course of the treatment (McGranahan and Swanton, 2015; Yang et al., 2017; Hong et al., 2018). This phenomenon was observed in a recent study which used single-cell DNA-sequencing to show that pre-existing resistant cells were selected for by neo-adjuvant chemotherapy in TNBC, leading to the development of therapeutic resistance (Kim et al., 2018; Hinohara and Polyak, 2019). Some of these resistant subpopulations in tumors may be caused in part via the presence of cells with BCSC and/or EMT characteristics, largely obtained through non-genetic means and thus particularly difficult to detect and/or target (Hong et al., 2018).

BCSCs are associated with therapy resistance and relapse. Compared with highly proliferative breast cancer cells, BCSCs are thought to remain in the G0 phase of the cell cycle for long periods of time (a quiescent state also known as dormancy) which likely contributes to their ability to resist chemotherapy and/or radiation damage (Allan et al., 2006). For example, Chang and colleagues compared the biopsies of patients' primary tumors before and after 12 weeks of treatment with neoadjuvant chemotherapy and found that the percentage of BCSCs (CD44^high^/CD24^low^) was increased after chemotherapy (Li et al., 2008). Similarly, Noguchi and colleagues also found that chemotherapy increased the percentage of BCSCs, however their results suggest that ALDH1-positivity as a marker of BCSCs was significantly more predictive than CD44^+^/CD24^−^ (Tanei et al., 2009). As early as the 1990s, and well before ALDH was associated with cancer stem cell phenotypes, it was known to be associated with chemoresistance due at least in part to its ability to metabolically inactivate chemotherapeutic agents such as cyclophosphamide (Mirkes et al., 1991). More recently, it has been shown that ALDH enhances breast cancer cell resistance to chemotherapy via up-regulation of many therapy-resistance proteins (p-glycoprotein, GSTpi, and/or CHK1) (Croker and Allan, 2012). In addition, BCSCs are reported to express high levels of ATP-binding cassette (ABC) transporter proteins, which protect cells from drug damage via efflux pumping mechanisms (Hirschmann-Jax et al., 2004).

The resistance of CSCs to radiation is largely due to the heightened ability of these cells to activate the DNA damage checkpoint, increasing the repair of DNA damage and decreasing resultant cell death (Rich, 2007). For example, the repair of radiation-induced DNA damage in mammospheres (enriched for breast cancer stem cells and their progenitors) is dramatically increased when compared with monolayer cultures (Phillips et al., 2006). Furthermore, several studies have demonstrated that BCSCs also more efficiently reduce intracellular reactive oxygen species (ROS) induced by ionizing radiation (Phillips et al., 2006; Diehn et al., 2009). ROS is a critical mediator of cell killing after exposure to ionizing radiation, and thus decreasing ROS can enhance the resistance of cancer cells to radiation (Riley, 1994).

As outlined above, a strong association exists between EMT and CSC phenotypes, and thus it is not surprising that the two phenotypes have been linked to resistance in the same context. In basal/HER2^+^ breast cancer cell lines, Menendez and colleagues showed that EMT-associated transcription factors (Snail2 and Slug) enhance resistance to trastuzumab via inducing a BCSC phenotype (CD44^+^CD24^−/low^) (Oliveras-Ferraros et al., 2012). In addition, recent studies also suggest that EMT may contribute to drug resistance directly. For example, it was found that EMT-associated markers, including Vimentin and MMP2, were increased in residual breast cancers after conventional therapy (Creighton et al., 2009), suggesting that breast cancer cells with molecular signatures associated with EMT may be more resistant to endocrine therapy (letrozole) or chemotherapy (docetaxel). Importantly, if EMT inhibits cancer cell proliferation (Vega et al., 2004; Mejlvang et al., 2007), this feature alone may increase chemotherapy resistance. In an elegant study in which cells undergoing an EMT were fate-mapped in a genetically engineered mouse model of breast cancer (MMTV-PyMT), Gao and colleagues demonstrated that mammary carcinoma cells that had undergone an EMT were much more resistant to chemotherapy than carcinoma cells that had not undergone an EMT, likely due to reduced proliferation, apoptotic tolerance and increased expression of chemoresistance-related genes in the EMT cells (Fischer et al., 2015). Similarly, it has been shown that Twist1 (a master regulator of EMT) is associated with multi-drug resistance in breast cancer, however, the mechanism by which Twist1 leads to resistance was not explored (Li et al., 2009). In a separate study, it was found that Twist can increase the transcription of ABC transporters, enabling efflux of drugs and an association with multidrug resistance (Saxena et al., 2011). Further, mathematical modeling of sequential therapy targeted toward either epithelial or mesenchymal tumor cells was shown to actually increase E/M plasticity leading to therapy resistance of breast cancer cells (Mathis et al., 2017). Modification of the therapeutic schedule to use alternating rather than sequential therapy was able to overcome this effect by killing both epithelial and mesenchymal cells and preventing phenotypic switching (Mathis et al., 2017). These data suggest that cells that have undergone an EMT or cells with enhanced plasticity may display heightened resistance to conventional cancer therapies, potentially through both active and passive mechanisms. As such, therapies that can target these plastic tumor cell populations may enhance therapeutic efficacy in patients who have failed one or more lines of conventional therapy.

## Mechanisms That Promote Cancer Cell Plasticity

Cancer cell plasticity can be regulated by numerous signaling pathways, and likely is a characteristic driven by the aggregate functions of multiple signaling pathways simultaneously. Below, we discuss the role of some of the pathways that appear to play very critical roles in the induction of plasticity in breast cancer by regulating CSC and EMT phenotypes. In addition to the following pathways, other pathways are also heavily implicated in both CSC and EMT cancer biology, including TGF-ß signaling, which is known to be critical for these processes. This pathway, as well as other pathways that we could not address due to space limitations of this review, have been extensively reviewed elsewhere (Wendt et al., 2009; Xu et al., 2009; Singh et al., 2015; Bellomo et al., 2016; Li et al., 2017).

### Mitogen-Activated Protein Kinase (MAPK) Pathway

The MAPK pathway is evolutionarily conserved and controls cell growth, proliferation, differentiation, migration, and apoptosis (Dhillon et al., 2007). Aberrant activation of MAPK is known to play a significant role in breast tumor onset and progression (Dhillon et al., 2007). This may in part be due to a role for MAPK signaling in the promotion of maintenance of CSC populations in tumors. Arteaga and colleagues found that loss of dual specificity phosphatase-4 (DUSP4), a negative regulator of the MAPK pathway, promoted cancer stem cell-like phenotypes in basal-like breast cancer (Balko et al., 2013). Similarly, activation of epidermal growth factor receptor (EGFR) leads to an expansion of CD44^+^/CD24^−^ populations in TNBC (which is heavily enriched in basal-like breast cancer) in a mitogen-activated protein kinase kinase/extracellular signal-regulated kinase (MEK/ERK) dependent manner (Wise and Zolkiewska, 2017). But MAPK regulation of CSCs is not limited to the TNBC and/or basal subtype. Indeed, our own group demonstrated that a developmental homeoprotein, SIX1, induces a CSC phenotype in luminal B breast cancer cells through induction of MAPK/ERK signaling (Iwanaga et al., 2012). MAPK signaling has also been implicated in the plasticity of cells in inflammatory breast cancer, where the MAPK interacting (Ser/Thr)-kinase (MNK) can activate NFκB signaling via increasing the expression of X-linked inhibitor of apoptosis, resulting in increased stem cell like characteristics as measured by ALDH expression (Evans et al., 2018).

In addition to its role in cancer cell stemness, MAPK signaling has also been shown to play a role in promoting EMT and EMP. For example, overexpression of RAS in human mammary epithelial cells, and resultant induction of MAPK signaling, results in an EMT and endows cells with stem and tumorigenic characteristics (Milsom et al., 2008). Further, constitutive activation of Raf-1 in MCF-7 cells lead to the development of distant metastases in xenograft models by promoting EMT (Leontovich et al., 2012). Numerous molecules promote EMT and EMP via activation of MAPK signaling. Examples include YB-1 (Evdokimova et al., 2009) as well as Doublecortin-like kinase 1 (DCLK1) (Liu et al., 2019), which can induce an EMT at least in part via activating MAPK signaling.

While these data suggest that cell autonomous control of MAPK signaling can promote cancer cell stemness and plasticity, MAPK signaling, and subsequent cancer cell plasticity, is also regulated by signaling molecules in the tumor microenvironment. Lijun Hao's group found that in breast cancer, stem cell factor (SCF) released by adipose-derived stem cells promoted an EMT phenotype (increased expression of N-cadherin, vimentin, and Twist and decreased expression of E-cadherin) and increased pulmonary metastasis in mouse models by downregulation of miR-20b. The authors determined that SCF-induced miR-20b downregulation was dependent on activation of the c-Kit/MAPK-p38/E2F1 signaling cascade (Xu et al., 2019). This finding suggests that EMT and cancer cell plasticity may not only be a cell autonomous characteristic but also may depend on the composition of the microenvironment surrounding a given cell.

### Phosphoinositide 3-Kinase (PI3K)/AKT/Mechanistic Target of Rapamycin (mTOR) Pathway

Aberrations in the PI3K/AKT/mTOR pathway are common genomic abnormalities in the majority of human cancers including breast cancer (Cancer Genome Atlas, 2012). Recent studies demonstrate that the PI3K/AKT/mTOR pathway plays an important role in breast cancer cell plasticity. For example, Wandosell and colleagues demonstrated that knockdown of AKT1 (and to a lesser extent AKT2) in the human triple negative breast cancer cell line MDA-MB-231 reduces CSC-like phenotypes and EMT characteristics in breast cancer cells, suggesting a reliance on flux through the PI3K/AKT pathway for maintenance of EMP and cancer cell stemness (Gargini et al., 2015). Similarly, Watson and colleagues showed that the inflammatory cytokine, oncostatin-M, mediates breast cancer cell stem and EMT characteristics via activation of PI3K signaling (West et al., 2014). Isolation of BCSCs (CD44^+^/CD24^−^/CD45^−^) from primary ERα-positive breast cancer followed by next generation sequencing- and microarray-based gene expression profiling clearly demonstrate that PIK3CA and other PI3K pathway genes are overexpressed in this population and the pathway is known to be involved in maintaining cancer stem cells in ER-positive breast cancer (Hardt et al., 2012). Intriguingly, while many pathways involved in plasticity are not mediated via genetic alterations, PI3K signaling alterations are often found to be due to mutations. Indeed, PIK3CA mutations are frequent in breast cancer, occurring in 28–47% of hormone receptor-positive breast cancers, 25% of HER2-positive breast cancers, and 8% of basal-like tumors (Stemke-Hale et al., 2008). The large percentage of hormone receptor positive tumors carrying PIK3CA mutations alludes to a particularly important role for this pathway in CSCs in this subtype of the disease.

### Signal Transducer and Activator of Transcription 3 (STAT3) Pathway

STAT3, a downstream effector of several receptor tyrosine kinases (RTKs) commonly activated by growth factors and cytokines, is persistently activated in all breast cancer subtypes (Walker et al., 2014; Banerjee and Resat, 2016). Constitutive STAT3 activation in breast cancer cells induces EMT and CSC properties. Compared with other breast cancer subtypes, STAT3 is most often associated with triple negative tumors, which are rich in cancer stem cells (Banerjee and Resat, 2016). However, some studies report that STAT3 is downstream of HER2 and may be associated with CSCs in this subtype of breast cancer also (Hartman et al., 2011; Chung et al., 2014). Therefore, it appears that STAT3 signaling is an important mediator of EMT and stemness across many genetically-distinct breast cancers.

In studying the relationship between STAT3 and CSCs, Polyak and colleagues found that the IL-6/JAK2/Stat3 pathway is preferentially active in CD44^+^CD24^−^ stem-enriched breast cancer cells, where it is required for their growth (Marotta et al., 2011). Additionally, work by the Slingerland group demonstrated that a VEGF/VEGFR2/STAT3 axis promotes breast and lung CSC self-renewal via upregulation of Myc and Sox2 (Zhao et al., 2015). In addition to stemness, STAT3 also regulates EMT. A study demonstrated that in breast cancer cells, phosphorylated STAT3 up-regulates the EMT associated protein, TWIST (Lo et al., 2007). Further, several studies have demonstrated that STAT3 up-regulates MMP2, MMP7 and MMP9 in breast cancer cell lines (Song et al., 2008; Yuan et al., 2008; Wang et al., 2012), proteins that are heavily associated with EMT. Additionally, through regulation of EMT/stemness, STAT3 may also play a role in regulating drug resistance, as it was shown that in human breast cancer, feedback activation of the IL6-STAT3 loop induced EMT and cancer stem cell features, leading to resistance to PI3K inhibitors (Yang et al., 2014).

### Wnt Pathway

Wnt signaling, which regulates cell polarity, proliferation, migration, survival, and maintenance of somatic stem cells, is very important in normal embryonic development (Clevers and Nusse, 2012) and its aberrant activation is involved in many malignant diseases, including breast cancer (Clevers and Nusse, 2012; Blagodatski et al., 2014). A growing body of evidence suggests that dysregulation of Wnt signaling promotes mammary tumor formation (Nusse and Varmus, 1982; Lane and Leder, 1997; Theodorou et al., 2007). In addition, Wnt signaling contributes to breast cancer progression at least in part due to increases in CSC and EMT phenotypes, suggesting that Wnt signaling is critical for cell plasticity. An example of this was shown by Varmus and colleagues, who found that expression of Wnt-1 in mammary glands of transgenic mice expands a population of basal-like cells which are enriched in stem like cells (Li et al., 2003). Similarly, Hong and colleagues showed that Wnt/β-catenin activity in BCSCs (ALDH1 positive) is significantly higher than in bulk cancer cells, and that blockade of Wnt/β-catenin signaling suppresses CSC-like phenotypes in a mouse model of breast cancer (Jang et al., 2015a). Interestingly, SOX9, which is an important pluripotency factor, was identified as a Wnt-target in intestinal crypts (Blache et al., 2004). In breast cancer, SOX9 enhanced T-cell factor 4 (TCF4) transcription and Wnt/β-catenin signaling (Wang et al., 2013). These studies highlight the possibility of a feedback loop between Wnt/β-catenin and SOX9 in promoting BCSCs. Finally, Wnt signaling may similarly promote EMT, as Weiss and colleagues demonstrated that the Wnt–Axin2–GSK3β cascade induces an EMT-like program via up-regulating Snail1 in breast cancer cells (Yook et al., 2006). Functionally these alterations in stemness and EMT may facilitate tumor progression, as data from Leyland-Jones's group demonstrated that breast cancer patients whose tumors had elevated Wnt/β-catenin signaling are more likely to develop lung and brain secondary metastases (Dey et al., 2013).

### The Hedgehog (Hh) Pathway

The Hh pathway is involved in embryonic mammary gland induction, development of ductal architecture and the differentiation that occurs prior to lactation (Lewis and Veltmaat, 2004). Emerging evidence suggests that dysregulation of Hh signaling is implicated in breast cancer development (Hatsell and Frost, 2007; Bhateja et al., 2019), though there is controversy around whether the primary role is in the tumor cells themselves or in the tumor microenvironment (Sun et al., 2014; Sims-Mourtada et al., 2015; Yang et al., 2016; Koike et al., 2017; Neelakantan et al., 2017). Multiple studies have linked Hh signaling to promotion and maintenance of CSC phenotypes in breast cancer. Wang and colleagues found that in human estrogen receptor positive breast cancer cells, estrogen promotes a CSC and EMT phenotype via activation of Gli1, a downstream effector of the Hh pathway (Sun et al., 2014). Similarly, Sims-Mourtada and colleagues found that Hh pathway activation mediates the activity of BCSCs and clonogenic re-growth of breast cancer cells after chemotherapy treatment (Sims-Mourtada et al., 2015). Consistent with this finding, recent studies showed that inhibition of the Hh pathway attenuates stem cell phenotypes such as CD44^+^/CD24^−^ cells and sphere forming capacity in breast cancer cell lines (Yang et al., 2016; Koike et al., 2017).

In addition to promoting stemness, Hh pathway activation can also promote EMT in breast cancer cells. For example, Tan and colleagues found that Twist1 and Snail (important EMT-associated transcription factors) are direct transcriptional targets of Gli1 (Kong et al., 2015). Similarly, Frost and colleagues found that Gli1 enhances breast cancer cell EMT and metastasis via up-regulation of MMP-11 (Kwon et al., 2011), suggesting that GLI proteins regulate numerous genes associated with EMT. Our lab recently demonstrated a key role for Hh/Gli pathway signaling in cellular plasticity in breast cancer cells as we showed that breast cancer cells that had undergone an oncogenic EMT could increase metastasis of neighboring cancer cells via both canonical and non-canonical paracrine-mediated activation of GLI activity (Neelakantan et al., 2017). These data suggest rapid alterations in plasticity and metastatic characteristics in response to signals that emanate from neighboring tumor cells, underscoring the critical nature of cell-cell crosstalk in inducing a plastic phenotype. Importantly, co-expression of GLI1 and two GLI1 targets, EGFR and Snail, are associated with worse outcome in breast cancer patients (Rudolph et al., 2018), further underscoring the clinical relevance of this pathway.

### Notch Pathway

The Notch signaling pathway, which is heavily associated with stemness, self-renewal, and differentiation during development, is essential for the development of multiple organ systems including mammary gland (Bolos et al., 2007). Activation of Notch signaling has been extensively linked to malignant progression in multiple solid cancer types, including breast cancer (Bigas and Espinosa, 2018). The breast tumorigenic ability of Notch has been known since the 1990s. Notch genes (Notch1 and Notch4), when expressed under the control of whey acidic protein (WAP) or mouse mammary tumor virus (MMTV) promoters in the mouse, result in the formation of mammary carcinoma (Jhappan et al., 1992; Gallahan et al., 1996; Dievart et al., 1999). Attempting to understand potential mechanisms through which Notch signaling may facilitate tumorigenesis, Tagliabue and colleagues found that Notch1 signaling equipped breast cancer cells with tumor-initiating cell properties due to HER2 gene amplification, and these effects were reduced after blockage of Notch signaling using either γ-secretase inhibition or Notch1-specific silencing (Magnifico et al., 2009). Multiple Notch family members may be involved in maintaining tumor-initiating potential in breast cancer cells, as Clarke and colleagues demonstrated that Notch4 has a more significant impact than Notch1 in BCSCs, and that Notch4 inhibition produces a more robust effect with a complete inhibition of tumor initiation (Harrison et al., 2010). Intriguingly, the same group demonstrated that estrogen increases BCSC activity by activating Notch signaling and showed that BCSCs induced by NOTCH signaling contribute to anti-estrogen resistance in human breast cells (Harrison et al., 2013; Simoes et al., 2015). In an attempt to understand regulation of Notch signaling in breast cancer, a recent study by our laboratory demonstrated that in both estrogen receptor positive and triple negative breast cancer, the miR-106b-25 miRNA cluster upregulates NOTCH1 through stabilizing the protein via direct repression of the E3 ubiquitin ligase NEDD4L. Further, we found that this upregulation of NOTCH1 was required for tumor initiating cell induction in multiple breast cancer cell lines (Guarnieri et al., 2018).

In addition to affecting cancer stem cell biology, Notch signaling is involved in the induction of EMT, again underscoring its role in mediating breast cancer cell plasticity. Slug and Snail, which are two critical EMT-associated transcription factors, have been shown to be regulated by Notch signaling in breast cancer cells (Chen et al., 2010; Shao et al., 2015). In addition, Karsan and colleagues showed that Slug is a direct target gene of Notch1 in breast cancer (Leong et al., 2007). In line with these findings, Suh and colleagues demonstrated that Notch2 up-regulates multiple EMT-associated markers including Twist, Snail1, Slug, Vimentin, and Zeb1 in basal type breast cancer cells (Lee et al., 2018). Clinical studies performed in breast cancer patients demonstrate that Notch signaling activation is associated with reduced overall survival and poor prognosis (Reedijk et al., 2005; Dickson et al., 2007), underscoring the importance of this signaling pathway for tumor progression in human breast cancer.

## Therapeutic Strategies for Targeting Breast Cancer Cell Plasticity

**Figure 2:**
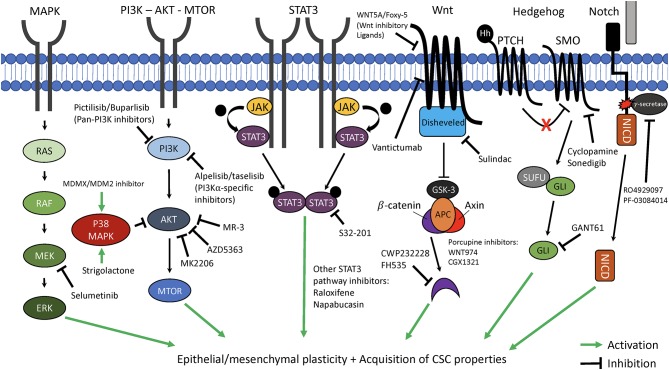
Therapeutic targeting of key pathways involved in cancer cell plasticity. The critical cellular pathways, mitogen-activated protein kinase (MAPK), phosphoinositide 3-kinase—protein kinase B—mammalian target of rapamycin (PI3K—AKT—MTOR), signal transducer and activator of transcription 3 (STAT3), Wnt, Hedgehog, and Notch each have been demonstrated to play key roles in promotion of epithelial-mesenchymal plasticity (EMP) and acquisition of cancer stem cell (CSC) properties. Highlighted are novel targeted therapeutics which can interfere with these pathways and may be able to suppress EMP and CSC characteristics of cancer cells. MEK/ERK, mitogen-activated protein kinase kinase/extracellular signal-regulated kinase; JAK, Janus-activated kinasel APC, adenomatosis polyposis coli; GSK3, Glycogen synthase kinase 3; PTCH/SMO, Patched/smoothened; SUFU, Suppressor of fused; NICD, Notch intracellular domain.

Because BCSCs and cells known to have undergone an EMT likely represent cancer cells with high degrees of plasticity, and because of the role of BCSCs and EMT in driving tumor initiation, invasion, metastasis, escape from the immune system, and resistance to chemo- and radiotherapy, it is often argued that targeting cancer stem cells and EMT may be the best way to therapeutically target phenotypically plastic cancer cells and improve patient survival. Over the last decade, numerous studies have focused on therapeutic strategies that target pathways involved in BCSC and/or EMT programs as a means to inhibit cancer cell plasticity and to reduce the overall ability of these cells to navigate the multiple environments encountered during the metastatic cascade (Figure 2).

### Targeting of the MAPK Pathway

Numerous small molecule compounds have been developed that target the MAPK pathway and based on the role of MAPK signaling in promoting EMP and stemness, inhibition of this pathway presents a logical therapeutic target. In line with this thought, emerging evidence indicates that selumetinib, a MEK1/2 inhibitor which has been used for phase I and II clinic trials in several kinds of malignant diseases (Bodoky et al., 2012; Hayes et al., 2012; Catalanotti et al., 2013; Ho et al., 2013; Jänne et al., 2013; Janne et al., 2015, 2016; Carvajal et al., 2014, 2015; Gupta et al., 2014; Brown et al., 2019), might be a novel therapeutic strategy for breast cancer. For example, Zolkiewska and colleagues found that selumetinib blocked EGF-induced expansion of CD44+/CD24– breast cancer stem cell associated populations (Wise and Zolkiewska, 2017). Similarly, the Ueno laboratory found that selumetinib inhibits the acquisition of breast cancer stem cell phenotypes and protects mice from lung metastasis after transplantation with TNBC cells (Bartholomeusz et al., 2015).

Paradoxically, other therapeutic approaches suggest that activation of the P38 MAPK pathway may have therapeutic benefit in breast cancer. Yarden and colleagues showed that Strigolactone was able to inhibit the growth of BCSCs via activation of P38 (Pollock et al., 2012). While this seemingly contradicts the role of MAPK signaling in CSC promotion, activation of P38 in this context actually suppressed AKT survival signaling, and the authors suggest that it is the suppression of PI3K/AKT signaling through P38 activation that is responsible for the observed inhibition of BCSC growth and thus provides a therapeutic benefit in this context (Pollock et al., 2012). Similarly, the Dong laboratory found that a dual-target murine double minute 2 (MDM2) and murine double minute X (MDMX) inhibitor suppresses EMT, migration, and invasion of TNBC cells through activation of the p38 MAPK pathway (Fan et al., 2019). Thus, while the MAPK pathway remains an attractive target for inhibiting cancer cell plasticity, the complex downstream signaling and cross-activation of other key signaling pathways by MAPK components suggests that targeting this pathway must be done thoughtfully in order to maximize therapeutic benefit.

### PI3K/AKT/mTOR Pathway

There are a number of different classes and isoforms of PI3Ks, and PI3Kα is the isoform predominantly mutated in cancer (Guerrero-Zotano et al., 2016). Currently, numerous compounds have been developed to inhibit PI3K signaling in breast cancer. Pictilisib and Buparlisib, which are orally available pan-PI3K inhibitors, have been studied in phase II or III clinic trials. Schmid and colleagues found that compared with use of the aromatase inhibitor anastrozole as a monotherapy, the combination of pictilisib and anastrozole significantly increases inhibition of tumor cell proliferation in luminal B primary breast cancer (Schmid et al., 2016). However, in the same year, another study found that in estrogen receptor-positive patients, the combination of pictisilib and fulvestrant did not increase progression free survival (PFS) compared to fulvestrant and placebo (Krop et al., 2016). In addition, dosing was limited by significant toxicities. Patients in the pictisilib + fulvestrant group showed a much higher rate of serious side effects than those in the fulvestrant + placebo group—these included pneumonitis, diarrhea, stomatitis, rash, and transaminitis, which led to dose reduction in 45% of cases and treatment discontinuation in 24% of patients (Krop et al., 2016). Compared with pictisilib, buparlisib is reported to have a better therapeutic effect with less associated toxicities (Mayer et al., 2014; Guerrero-Zotano et al., 2016). Baselga and colleagues found that Buparlisib plus fulvestrant significantly improved progression free survival (PFS) by 1.9 months (6.9 vs. 5.0 months, *p* < 0.001) in patients with hormone receptor-positive, human epidermal growth factor receptor 2-negative advanced breast cancer (Baselga et al., 2017).

Alpelisib and taselisib are two PI3Kα specific inhibitors which are ideal drug candidates for patients with PIK3CA mutations. Studies demonstrate that these two drugs, either used as single agent or combined with endocrine therapy, showed preferential therapeutic effects against breast tumors harboring PIK3CA mutations, and as such, these drugs represent a means for personalized, tumor specific therapy (de Jonge et al., 2014; Mayer et al., 2017; Tamura et al., 2018; Baird et al., 2019; Saura et al., 2019). In addition to targeting PI3K itself, other therapeutic approaches have attempted to target the downstream target of PI3K, AKT. MK-2206, and AZD5363, two inhibitors of AKT, exhibited promising activity against breast cancer cells in preclinical studies (Crafter et al., 2015; Ribas et al., 2015; Choi et al., 2016; Baek et al., 2018; Chen et al., 2018). However, these drugs showed limited clinical efficacy in clinical trials (Kalinsky et al., 2018; Turner et al., 2019; Xing et al., 2019). A distinct attempt to target AKT activity was made by Chen and colleagues, who recently discovered that a natural methoxylated analog of resveratrol, 3,5,4′-trimethoxystilbene (MR-3), can block EMT and the invasion of breast cancer cells via restoring GSK3β activity and inhibiting the phosphorylation of AKT (Tsai et al., 2013). However, the effects of this compound have not yet been tested clinically. While PI3K and AKT remain promising drug targets for selective inhibition/elimination of phenotypically plastic cancer cells, high toxicity and mixed efficacy for many candidate therapeutics in clinical trials indicates a need for further research to better characterize the druggability of this key signaling pathway.

### STAT3 Pathway

In breast cancer, most STAT3 pathway inhibitors are still in the preclinical phase of development, but represent a promising category of therapeutics due to the role of STAT3 in promoting cancer cell plasticity. Sun and colleagues found that pharmacological inhibition of STAT3 with S32-201 reduced breast cancer cell EMT and stem-like properties. In addition, disruption of the IL6-STAT3 signaling pathway can overcome resistance to PI3K inhibitors, suggesting that combined blockade of STAT3 and PI3K signaling might be a more efficient therapeutic strategy for breast cancer (Yang et al., 2014). Lin and colleagues found that Raloxifene, a selective estrogen receptor modulator which was approved for reducing the risk of invasive breast cancer, attenuates STAT3 phosphorylation and transcriptional activity via inhibiting IL-6/GP130 interaction in various cancer (including breast cancer) cell lines (Shi et al., 2017), suggesting that this inhibitor may work by influencing numerous critical pathways in hormone positive breast cancer cells. Strikingly, a phase Ib/II study showed that napabucasin, a first-in-class cancer stemness inhibitor that targets the STAT3 pathway, when given with weekly paclitaxel treatments, has shown promising effects in metastatic TNBC patients who have progressed on taxane-based treatment regimens (Becerra et al., 2016). Therefore, while we are just beginning to understand effective means of targeting STAT3, this may present a novel means for inhibiting cancer cell plasticity and associated tumor progression.

### Wnt Pathway

Wnt pathway inhibitors have been an area of active investigation for many years, but have often proven difficult to use due to associated toxicities (particularly affecting the GI tract). Currently, Vantictumab, a first-in-class antibody that inhibits canonical Wnt signaling by blocking five Frizzled receptors (1, 2, 5, 7, 8) (Ram Makena et al., 2019) is being used in combination with paclitaxel in phase 1b clinical studies in patients with metastatic HER2-negative breast cancer (Mita et al., 2016). WNT5A is a WNT inhibitory ligand, and Foxy-5, a WNT5A mimicking peptide, has been shown to reduce metastatic spread of WNT5A-low breast cancer cells in mouse models (Safholm et al., 2008; Canesin et al., 2017). Currently, a phase 1 clinic study is ongoing to evaluate the safety, tolerability, and pharmacokinetic profiles of Foxy-5 in patients with metastatic breast, colorectal, or prostate cancer (Soerensen et al., 2014). Numerous groups continue to investigate novel Wnt inhibitors that may have a more tolerable side effect profile than earlier ones tested. For example, CWP232228, the Wnt/beta-catenin inhibitor which blocks β-catenin binding to TCF in the nucleus, inhibits proliferation and activity of BCSCs (Jang et al., 2015b) and treatment with CWP232228 after tail vein injection of 4T1 mammary carcinoma cells decreased metastatic burden and increased overall survival in pre-clinical studies (Jang et al., 2015b). Similarly, Hong and colleagues found that FH535, another β-catenin/TCF inhibitor, significantly suppressed tumor sphere formation and the CD44^+^/CD24^−^ BCSC subpopulation in mouse breast cancer cells (Jang et al., 2015a).

One class of drugs that has recently received a lot of attention are Porcupine inhibitors. Porcupine is an acyltransferase which is involved in enabling secretion of all Wnt ligands, and thus represents an ideal drug candidate for targeting the Wnt pathway (Solzak et al., 2017). WNT974, a novel small molecule Porcupine inhibitor, was shown to reduce lung metastatic burden and increase survival when combined with the pan-PI3K inhibitor buparlisib in triple negative breast cancer PDX models (Solzak et al., 2017). WNT974 and CGX1321, another small molecule Porcupine inhibitor, are both currently in early stage clinical trials in advanced solid tumors^1^^,^ ^2^. Other known drugs may also exhibit inhibitory effects on the Wnt pathway such as Sulindac, a non-steroidal anti-inflammatory drug (NSAID), which was shown be able to inhibit Wnt signaling by binding the PDZ domain of disheveled (DVL1) (Lee et al., 2009). Importantly, Yang and colleagues found that Sulindac inhibits cell proliferation via downregulation of Wnt signaling in breast, lung and colon cancer cells (Han et al., 2008). As more data is amassed and as more clinical trials aimed at targeting the Wnt pathway complete, we will gain a better understanding of the therapeutic efficacy of inhibition of the Wnt pathway in preventing tumor progression.

### Hh Pathway

Compared with other signaling pathways, the strategies targeting the Hh pathway are more diverse. The Hh pathway can be inhibited via blocking Hh ligands, receptors (such as SMO), or downstream transcription factors (GLI) (Bhateja et al., 2019). A monoclonal antibody against Hh ligands (5E1) was shown to inhibit breast cancer growth and metastasis in mouse models (O'Toole et al., 2011). Cyclopamine, a naturally occurring chemical with a high affinity for SMO, can be used to block Hh pathway signaling and can reduce breast cancer cell viability (Mukherjee et al., 2006). However, the low potency and poor solubility of cyclopamine has limited its clinical use. Another SMO inhibitor, sonidegib, which is FDA-approved for the treatment of advanced and metastatic basal cell carcinoma, was evaluated in phase I and II clinical trials for patients with breast cancer (Stathis et al., 2017; Ruiz-Borrego et al., 2019). Unfortunately, it has been less efficacious in breast cancer, despite evidence for activated Hh signaling (Stathis et al., 2017). Our previous studies showed that GLI signaling is activated downstream of EMT transcription factors in both SMO-dependent and SMO-independent manners (Neelakantan et al., 2017), providing a potential explanation for why SMO inhibitors are not efficacious in breast cancers with evidence of activated Hh signaling. Instead, we found that GANT61, a GLI antagonist which interferes with GLI translocation to the nucleus, is more efficacious in PDX models of breast cancer than SMO inhibitors (Neelakantan et al., 2017). Consistent with our results, Bei and colleagues found that GANT61 inhibited Hh pathway activity and breast cancer cell survival more effectively than GDC-0449 (a SMO inhibitor) (Benvenuto et al., 2016). Furthermore, in a mouse breast cancer model (TUBO cells), GANT61 caused complete tumor regression in 80% of mice, and these mice remained tumor free for up to 30 weeks (Benvenuto et al., 2016).

### Notch Pathway

γ-Secretase is a membrane-embedded aspartyl protease that cleaves the Notch receptor and results in the release and translocation of its intracellular domain into the nucleus and subsequent activation of target genes (Lu et al., 2014). Due to its important role in Notch pathway activation, to date, many different γ-secretase inhibitors have been evaluated and they exhibit promising results (Kontomanolis et al., 2018). Strikingly, several γ-secretase inhibitors were used in breast cancer clinic trails. For example, RO4929097 was recently used in a phase I study in patients with refractory metastatic or locally advanced solid tumors, including breast cancer and showed excellent tolerance (Tolcher et al., 2012). Subsequently another phase I study in patients with advanced solid tumors, including breast cancer, showed that RO4929097 and gemcitabine can be safely combined and 22.22% of patients achieving a partial response or stable disease more than 3 months after the combined treatment (Richter et al., 2013). A phase I clinical trial of another γ-secretase inhibitor (PF-03084014) in combination with docetaxel in patients with metastatic TNBC is ongoing. At present, the combination treatment is generally well-tolerated, and 16% of patients treated achieved a partial response (Curigliano et al., 2015; Locatelli et al., 2017). These studies suggest that use of γ-secretase inhibitors may be an effective and well-tolerated way to inhibit the Notch signaling pathway and to subsequently treat metastatic or locally advanced cancers.

## Conclusion

In summary, due to the adaptability afforded by cellular plasticity, plastic breast cancer cells gain a fitness advantage during tumor progression, enabling them to adjust to an unfavorable microenvironment, evade immune attack, and spread from the primary tumor to a metastatic site. Further, such plasticity can enable escape from toxic effects of anticancer drugs. As a result, plasticity programs lead to the poor prognosis observed in patients with breast and other cancers. Targeting plasticity represents a promising therapeutic strategy to repress breast cancer metastasis and overcome therapy resistance and promote tumor regression.

## Author Contributions

DK: design and co-wrote draft manuscript. CH: design, co-wrote manuscript, editing, and drawing figures. HF: design, editing, and approval of final version.

## Conflict of Interest

The authors declare that the research was conducted in the absence of any commercial or financial relationships that could be construed as a potential conflict of interest.

## Footnotes

^1^*CGX1321 in Subjects With Advanced Solid Tumors and CGX1321 With Pembrolizumab in Subjects With Advanced GI Tumors (Keynote 596)*. (2016) Available online at: https://clinicaltrials.gov/ct2/show/NCT02675946 (accessed January 25, 2020).

^2^*A Phase II Trial Evaluating WNT974 in Patients With Metastatic Head and Neck Squamous Cell Carcinoma*. (2016) Available online at: https://clinicaltrials.gov/ct2/show/study/NCT02649530 (accessed January 25, 2020).

## Abbreviations

EMT: epithelial-to-mesenchymal transition
MET: mesenchymal to epithelial transition
CSC: cancer stem cells
BCSCs: Breast cancer stem cells
ALDH1: Aldehyde dehydrogenase1
NOD/SCID: nonobese diabetic/severe combined immunodeficient
TNBC: triple-negative breast cancer
ECM: extracellular matrix
MMPs: matrix metalloproteinases
TGF-ß: transforming growth factor ß
CTC: circulating tumor cells
FLPs: filopodium-like protrusions
DTCs: disseminated tumor cells
LOXL2: Lysyl oxidase-like 2
iPSCs: induced pluripotent stem cells
E/M: epithelial/mesenchymal
Tregs: regulatory T cells
PD-L1: programmed death-ligand-1
TAMs: tumor-associated macrophages
ABC: ATP-binding cassette
ROS: reactive oxygen species
MR-3: 3,5,4′-trimethoxystilbene
MAPK: Mitogen-activated protein kinase
EGFR: epidermal growth factor receptor
MEK/ERK: mitogen-activated protein kinase kinase/extracellular signal-regulated kinase
MNK: MAPK interacting (Ser/Thr)-kinase
PI3K: phosphoinositide 3-kinase
AKT/PKB: protein kinase B
mTOR: mechanistic target of rapamycin
STAT3: signal transducer and activator of transcription 3
TCF4: T-cell factor 4
WAP: whey acidic protein
MMTV: mouse mammary tumor virus
PFS: progression free survival
NSAID: non-steroidal anti-inflammatory drug
DVL1: disheveled1
FDA: food and drug administration.
